# Allergen Stability in Food Allergy: A Clinician’s Perspective

**DOI:** 10.1007/s11882-023-01107-9

**Published:** 2023-09-04

**Authors:** Larissa Koidl, Salvatore Alessio Gentile, Eva Untersmayr

**Affiliations:** Institute of Pathophysiology and Allergy Research, Center for Pathophysiology, Infectiology and Immunology, Medical University of Vienna, Waehringer Guertel 18-20, E3Q, 1090 Vienna, Austria

**Keywords:** Allergen stability, Food processing, Allergen digestion, Food allergy, Allergenicity

## Abstract

**Purpose of Review:**

The globally rising food allergy prevalence is associated with the urgent need for new disease prevention methods, efficient treatment, and reliable risk assessment methods for characterization of food allergens. Due to inter-individual variations in the digestive system, food allergens are degraded to a different extent in each person. Food processing also influences allergen digestion.

**Recent Findings:**

In this review, we provide an overview of the digestive system with focus on relevance for food allergy. Main food proteins causing allergic reactions are evaluated, and the combined role of food processing and digestion for allergen stability is highlighted. Finally, clinical implications of this knowledge are discussed.

**Summary:**

Recent literature shows that allergen digestibility is dependent on food processing, digestive conditions, and food matrix. Digestion affects proteins allergenicity. It is currently not possible to predict the immunogenicity of allergens solely based on protein stability.

## Introduction

The aim of this review is to summarize recent findings on allergen stability for proteins and carbohydrates relevant in food allergy. We report about recent knowledge advances on processing and digestion, and their impact on allergenicity, and discuss the relevance and implications of allergen stability for clinical practice.

### Epidemiology, Management, and Burden of Food Allergy 

The calculated prevalence for food allergy is varying between studies and surveys, with estimations depending on factors such as geography and study design/selection criteria (self-reported, physician-confirmed, IgE-mediated, non-IgE-mediated). The general consensus is that the prevalence of food allergy is rising worldwide, especially in urbanized areas. Roughly, up to 10% (with big variation depending on geography) of the population in industrialized countries is estimated to be affected by food allergy [1–4].

As food allergy is relevant to a significant part of the global population and numbers seem to be still rising, strategies for prevention, diagnosis, treatment, and management are urgently needed. Although oral immunotherapy (OIT) for peanut allergy is available and OIT for other allergens are performed in specialized clinical centers with or without support by biologicals, the best strategy in food allergy management is still avoidance of the causative trigger [5, 6]. This is associated with a negative impact on the quality of life of food allergic patients, especially for patients with multiple or severe allergies. Food allergy also increases direct and indirect economic burden of affected patients [7, 8].

The identification and characterization of food allergens as well as understanding the mechanisms associated with an allergic reaction are necessary for further advancing diagnosis, management, treatment, and prevention of food allergy.

The stability of allergens (i.e., their ability to maintain their structural integrity and allergenic property) in the context of processing and digestion is a key aspect in characterizing an allergen [9, 10]. Both for primary food allergens or for proteins cross-reactive to inhalant allergens (e.g., PR-10 proteins), the stability of the allergens against processing methods and resistance to digestion seem to impact on the allergenicity, which influences both, the sensitization capability and the allergy eliciting potential.

## Literature Search

The literature search for Chapter 1 (Introduction) and Chapter 3 (Processing and Digestion of Foods — an Overview) was conducted in May 2023 on PubMed. For the introductory sections, original research and reviews were equally considered and publications from the last 5 years (2018–2023) were preferably cited.

Literature search for Chapter 4 (Allergen-Specific Stability) was conducted in May 2023 on PubMed using the following keywords: (allergen AND stability) OR (allergen AND digestion) OR (allergen AND pepsin) OR (allergen AND food processing), Filters: publication date 2018–2023. Overall, 1863 manuscripts were identified. For this chapter, special focus was given to original research articles. Articles were screened by title and abstract, and 295 relevant articles were included for a detailed literature screening. Additional publications suggested by the recommendation algorithms of PubMed and Sciwheel were also considered and included if relevant.

## Processing and Digestion of Foods — An Overview

### Food Processing

Most food and the contained allergens in the food are processed with various methods before they reach the consumer. Examples are heating/thermal processing, chemical modification, enzymatic treatment, fermentation, microwaving, irradiation, and modification of food matrix, and recently, also, cold plasma processing was introduced [10–13]. Table 1 shows an overview summarizing the processing methods currently applied in food industry.

**Table 1:** Overview: food processing methods (expanded from [14–17])

Classification	Processing method	References
Thermal	Baking, steaming, boiling, roasting	[18–22]
	Pasteurization, ultra-high-temperature treatment	[23, 24]
	Drying, smoking	
	Maillard reaction/glycation	[25–27]
Non-thermal [28]	Pressure	[29, 30]
	Microwave	[11]
	Cold/atmospheric plasma	[31–33]
	Freezing	[34]
	Drying (sublimation, radiation, …)	
	Ultrasound	[35–37]
	Irradiation/UV	[38]
	Pulsed electric field	
	Membrane processing	
	Enzymatic treatment (hydrolysis, lipolysis)	[39, 40]
	Fermentation	[41, 42]
Combination methods	Autoclaving: heat + pressure Drying (evaporation)	[43–45]
Additional considerations	- Impact of food matrix - Food additives - Impact of extraction method in research	- [20, 46] - - [47]

Processing alters the allergen by either destroying or modifying the structure, and consequently changes the susceptibility of the allergen to digestive enzymes. Moreover, the antibody recognition site of food proteins, the epitopes, might be exposed or hidden. This can result in a changed allergenicity. A lot of research is performed on the topic of food processing to reduce the allergenic potential of food to increase food safety and novel processing methods are developed with the aim to reduce the allergenicity of food proteins.

### The Digestive System and Food Allergens

Upon exposure to a novel food protein, the potential allergen can induce sensitization in not yet allergic persons if oral tolerance is impaired. The term “food allergy” encompasses a wide variety of different diseases, which have an impaired tolerance development towards food contents and an adverse immune reaction in common. The underlying mechanisms can be IgE-mediated or non-IgE-mediated. Food allergies can cause reaction along the digestive tract, but also in non-digestive organs, such as the skin (e.g., urticaria, angioedema), lung, and the cardiovascular system [48].

Food and its allergenic proteins are transported through the digestive organs of the body. The digestive process starts in the oral cavity where food is comminuted physically by chewing and pre-digested via a diverse repertoire of salivary enzymes. Saliva has a pH of 6 to 7 with buffering capabilities and consists mostly of water containing electrolytes and proteins [49]. Its production and composition undergo age-related changes [50]. A digestive enzyme in saliva, α-amylase, enables an initial breakdown of starches into smaller sugars. The lingual lipase initiates the lipid digestion by breaking down triglycerides. Besides digestive enzymes, saliva also contains mucins. The secretory mucin glycoprotein MUC5B is of high molecular weight and the primary gel-forming mucin in the oral cavity. The smaller MUC7 functions less as lubricant, but acts as a protection against bacteria [51]. Other mucins found in the oral cavity are the epithelial MUC1 and low levels of MUC4. Oral mucins are able to interact with allergenic food proteins such as caseins and whey proteins, by aggregation and emulsification, therefore changing the protein characteristics [52]. Saliva also contains antimicrobial proteins (lysozyme, peroxidases, lactoferrin, cystatins, histatins, statherin, etc.), immunoglobulins (especially secretory IgA and IgG) [53], and other compounds. Approximately 10^6^ leukocytes are found in oral rinsing solution, with neutrophils being present in highest numbers, followed by mononuclear cells and relatively small numbers of basophils and eosinophils [54]. A first sampling of food antigens takes place in the tonsils. The first breakdown of food in the oral cavity leads to size reduction and structural changes of the ingested food. Oral digestion degrades proteins, carbohydrates, and lipids and influences the gastric and intestinal digestion further downstream, as it exposes cleaving sites of food content to enzymes [17].

The orally pre-digested and macerated food is transported via the esophagus into the stomach, where gastric digestion takes place. A recent study suggested a division of the stomach into two major functional sections based on proteomics experiments. The cardia, fundus, lesser curvature, and greater curvature were allocated to the proximal section. The major task attributed to the proximal section was secretion of gastric juices, maintaining the acidic environment (pH < 2) and digestive functionality (high expression of pepsin-related gene PGA3). The angular incisures, antrum, and pylorus are part of the distal section. The reported tasks of this section was digestion of food, contraction, and maintaining the mucus barrier to prevent autodigestion [55, 56].

Gastric acid is produced by combination of luminal chloride anions (Cl^−^) with protons (H^+^), which are secreted by the H^+^-K^+^-ATPase found on the luminal membrane of parietal cells (located in the corpus glands). The highly acidic gastric juice in the stomach not only eliminates pathogenic microbes ingested with food but also denatures proteins and makes them more susceptible to the proteolytic enzyme pepsin. Gastrin (produced by G cells in the antral glands), histamine, acetylcholine, ghrelin, somatostatin (inhibitory) and glucagon-like peptide 1 (inhibitory), the vagus nerve, and other mechanisms regulate the acid production [56–58]. The main digestive enzyme of the gastric juices is pepsin, which cleaves proteins into smaller peptides and amino acids. Pepsin is produced by the chief cells of the stomach lining, secreting the inactive pepsinogen. HCl activates pepsinogen to pepsin. The optimum pH for pepsin activity is at pH 1.5 to 2.5 [59, 60]. Another digestive enzyme found in the stomach is the gastric lipase [17]. In the stomach the mucins MUC5AC and MUC6 are secreted, while MUC1 is membrane-bound [61]. A special feature of the gastric mucus is the double-layered structure, which is only found here and in the colon [62].

The gastric digest (called chyme) is further transported into the small intestine. The small intestine is divided loosely into 3 sections, the most proximal section being the duodenum, followed by the jejunum in the middle, and the distal ileum. In the duodenum, the gastric acid is neutralized by bicarbonates (released mainly by the pancreas) leading to an inactivation of pepsin by the higher pH levels (approximately pH 6) [60, 63]. The pH of the small intestine gradually increases to around 7.1 in the mid sections and rises further to approximately 7.4 in the ileum [63]. The tasks of the small intestine are digestion, and absorption of nutrients while maintaining barrier function against unwanted luminal contents. For this purpose, small intestinal and especially duodenal cells can produce a repertoire of enzymes, transporters, receptors, hormones, and other proteins, peptides, and bioactive mediators, which form the chemosensory system [64].

Protein digesting endopeptidases and exopeptidases produced by the pancreas are present in the duodenum. The pancreatic enzymes trypsin, chymotrypsin, and elastase belong to the former group, while carboxypeptidase A and B belong to the latter group [65••]. Carbohydrates are degraded by the pancreatic α-amylase, which has a pH-dependent N-glycan-specific binding activity. It binds to high-mannose type oligosaccharides at neutral pH and both complex and high-mannose type oligosaccharides at acidic pH [66].

Absorptive enterocytes possess brush border enzymes, which digest peptides, disaccharides, and other micronutrients into absorbable monomers (amino acids and monosaccharides) or small oligopeptides [64] and play a role in the surveillance of the luminal environment. These brush border enzymes include carbonic anhydrase (for acid sensing), intestinal alkaline phosphatase (IAP, for ATP sensing), adenosine and deaminase for adenosine sensing, and peptidases such as dipeptidyl peptidase 4 (DPP4) for rapid degradation of gut hormones (glucagon-like peptide 1 and 2), gastric inhibitory polypeptide, vasoactive intestinal polypeptide (VIP), ACE and ACE2 in high quantities, aminopeptidases, and dipeptidases and tripeptidases [64, 65••]. The digestive brush border enzymes are important for the final digestion of nutrients before absorption [67]. They are also relevant in food allergy, as it was demonstrated in tree nut allergy [20]. Major mucins found in the small intestine are the secretory MUC2 and the membrane-associated MUC13 and MUC17. MUC6, which is found in the stomach, is also expressed in the duodenum. As in other mucosal tissues, the type and amount of mucus-expressed glycans is subject to changes. The intestinal mucins are produced by goblet cells [62, 68, 69].

The final stage of digestion is taking place in the large intestine by the microbiome, which ferments luminal content. Fermentation by microbiota and production of microbial metabolites is an important topic, which we reviewed in the past also with its relevance for food allergy (reviewed in [70]). While human enzymes have limited ability to degrade glycans, the gut microbiota play a pivotal role in glycan degradation. The gut microbiota possess a wide range of glycan-degrading enzymes, allowing them to break down and utilize various dietary and host-derived glycans [71]. These microbial enzymes can cleave the glycosidic linkages present in glycans, releasing smaller sugar units that can be metabolized by the gut bacteria for energy and other purposes. Glycosylated proteins are provided to the bacteria not only via ingested food but also in form of heavily glycosylated mucus proteins. The dual-layer mucus is habitat and food source for the large intestinal microbiota. *Akkermansia municiphila*, a mucin-degrader, is associated with many health-related outcomes [72], but also other microbiota use mucins as energy source [73]. Membrane-bound mucins found in the colon are MUC3, MUC4, MUC12, MUC13, MUC15, MUC17, and MUC21 [68]. The major secretory mucin of the colon is MUC2. The type of mucins produced as well as the amounts may change with age and during pathological processes [62, 74–77].

### In Vitro Versus In Vivo Digestion

The individual digestive capacity of a person leads to varying extend of protein digestion, with influencing factors being age (e.g., early life versus adults versus elderly), medication (e.g., proton-pump-inhibitors), individual enzyme production, and the personal microbiome [65••]. These individual aspects should be considered when designing assays simulating gastrointestinal digestion for protein characterization.

Protein digestibility depends on the experimental conditions, such as duration, pH levels, and enzymatic activity (type and concentration of the involved enzymes) [78]. This is not only observed for in vitro experiments, but differences in the in vivo conditions in the stomach of humans and also mice revealed that higher pH in the stomach resulted in a reduced digestion of allergen proteins and a higher susceptibility to sensitizations and allergic reactions in already sensitized organisms [79, 80]. In line, support of gastric digestion with enzyme supplementation protected against sensitization and allergic reactions [81]. Therefore, it remains highly relevant to evaluate the role of gastric protein digestion as an important physiological barrier and gatekeeper in food allergy.

In 2021, the EFSA published an update of the “GMO Panel guidance document on allergenicity of genetically modified (GM) plants” stating that the classical pepsin resistance test is probably not sufficient for the assessment of allergenicity of new proteins in genetically modified plants. The use of advanced in vitro digestion tests was suggested, which still need to be evaluated before implementation. In the statement, the necessity for more reliable prediction of what happens to proteins in the gastrointestinal tract and how they interact with human cells is summarized [82]. Recently, a protocol for simultaneously testing the resistance of multiple allergens against intestinal digestion in 96-well format was described. The authors tested several allergens with different pH levels and enzyme concentrations giving a good overview on allergen-specific digestion conditions [83••].

**Fig. 1:**
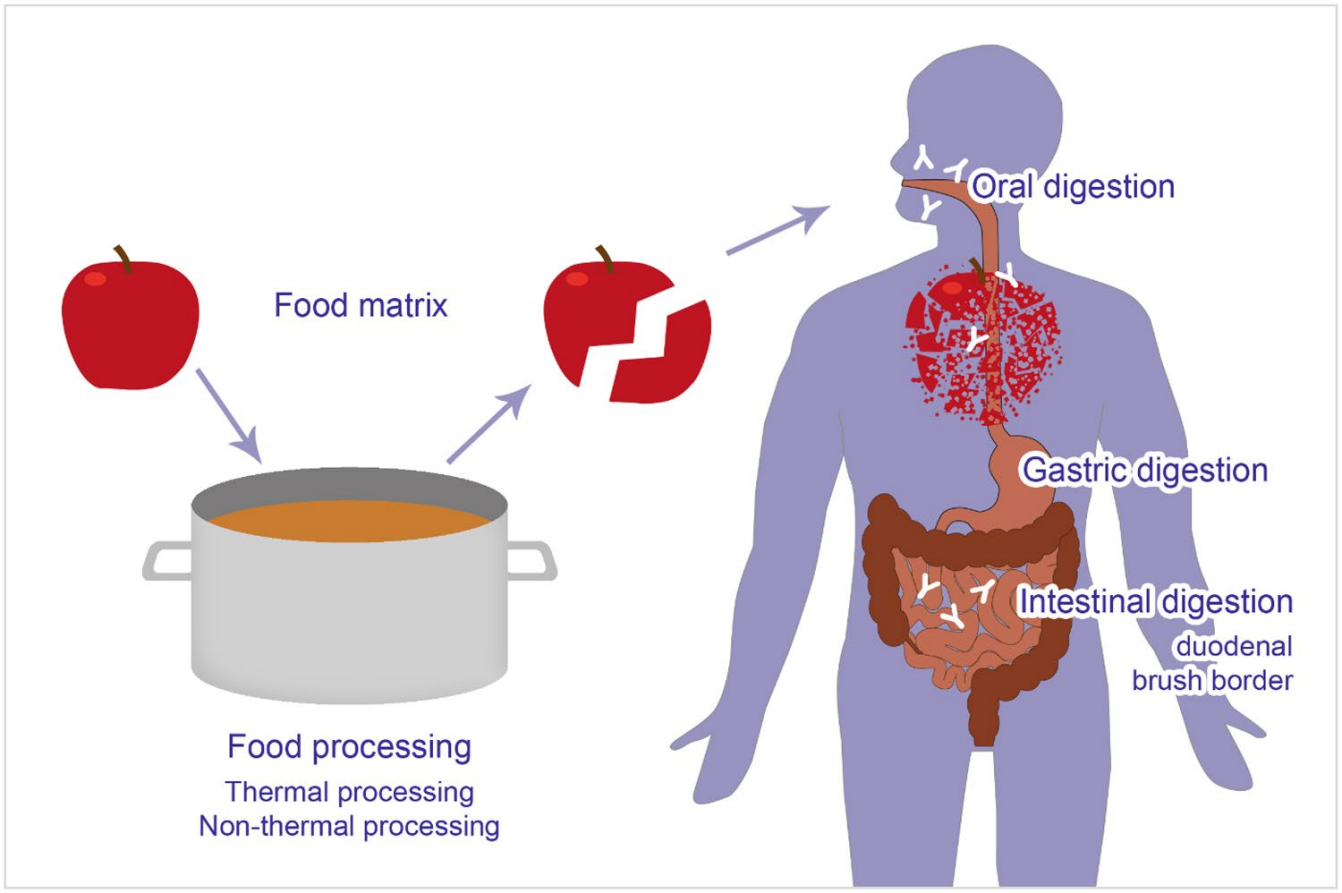
Factors influencing allergen stability. The stability of food allergens is determined during food processing, with various processing methods (thermal, non-thermal processing) having different impact on the stability. Moreover, the individual function of the oro-gastrointestinal tract influences protein digestion leading to an altered degree of allergen fragmentation. Moreover, the food matrix may also exert impact on the allergen stability

Besides digestion along the gastrointestinal tract, including the oral cavity, the food matrix, the extraction method, and the pre-digestive processing should also be considered, as these factors are highly relevant for allergen stability. Figure 1 depicts a short summary of factors influencing allergen stability. Standardized protocols, such as the INFOGEST protocol [84•], are needed to improve comparability between studies.

## Allergen-Specific Stability

In our previous manuscript on the topic of allergen stability [9], we have focused on the gastrointestinal tract and its function in allergen stability. The aim of the current review is to summarize novel findings on the topic of allergen stability with specific focus on food allergens. Most of the reviewed publications reported about the allergen stability under specific processing and digestion conditions.

We have selected the five main food allergens cow milk, egg, peanut, shellfish, and fish, due to available knowledge in context of allergen stability. These allergens are also the most common allergens in infants, children, and/or adults [85]. Additionally, we have added a section on carbohydrate/glycan allergens.

### Cow Milk Allergens

When breastfeeding is not possible, infants receive infant formula. For this reason, cow milk allergy is the earliest food allergy that arises in humans. In the USA, the prevalence of cow milk allergy in infants is estimated to be around 2–3%, making it the most common allergy among infants and young children [86]. Therefore, research on cow milk processing to reduce its sensitization potential and allergenicity, as well as the fate of the allergens during (infant) digestion, are highly relevant.

Milk contains approximately 32 g protein per liter, and its proteins can be divided into two groups: the insoluble casein fraction, which makes up the majority of milk proteins (around 80%), and the soluble whey proteins (about 20%) [87]. Allergies can arise against both caseins and whey proteins. The milk proteins show different degrees of stability against digestion with β-lactoglobulins (Bos d 5) being reported to be more stable against digestion than the other proteins and caseins [24, 43, 83••].

The stability of milk proteins can be modified by different processing methods. In a study comparing pasteurized milk with ultra-heat-treated milk and dried skim milk [24], the authors reported that the main allergens in this three differently processed milk displayed different resistance to gastrointestinal digestion. Caseins in pasteurized milk and ultra-heat-treated milk were rapidly digested in the gastric digestion, while the caseins in ultra-heat-treated milk showed a higher resistance to gastric digestion. On the other hand, α-lactalbumin (Bos d 4) and β-lactoglobulin in pasteurized and dried skim milk showed a higher resistance to gastric enzymes compared to the same proteins found in ultra-heat-treated milk. The authors also showed that the peptides from the digests were further broken down as they passed through the intestinal epithelium using intestines of rats [24]. Thus, the processing method has to be considered when evaluating the stability of milk proteins.

Another type of milk processing to reduce the allergenic potential of milk is “baked milk.” Dry heating results in structural changes of milk proteins not only because of the heat itself but also due to glycation-induced changes. Baked milk displays an altered digestibility and immunoreactivity. While unheated samples showed higher numbers of specific IgE binding epitopes compared to the baked samples, no difference in the number of T-cell epitopes was observed between the two groups. Furthermore, the transepithelial shuttling of the peptides was simulated on Caco-2 cells and glucosyl lysine and lactosyl-lysine-modified peptides were the preferably transported glycated peptides [18]. Baked milk was already successfully applied in clinical studies in milk allergic children and increased tolerance towards other milk products [88].

Autoclaving (a combination and thermal and pressure-based processing) was reported to increase protein fragmentation and the digestion rate. Furthermore, it reduced the IgE-binding capacity of milk proteins [43].

### Egg Allergens

Egg is another important allergen source. The major egg allergens are found in egg white, namely, ovomucoid, ovalbumin (OVA), ovotransferrin, and lysozyme. Α-livetin and lipoprotein YGP42 are two minor allergens found in egg yolk. Eggs are of high nutritional value and are a globally consumed. In total, around 30% of eggs are consumed in processed form [89]. Mostashri et al. recently reviewed the impact of processing on the allergenicity of egg proteins in detail [90]. Therefore, we will focus on the digestion of egg proteins.

Digestion of egg proteins depends on the experimental conditions. Simulating infant gastrointestinal digestion was not able to completely digest egg proteins, neither for isolated proteins nor in food matrix [91]. When comparing different fractions of heated and hydrolyzed egg whites, the egg peptide fraction with less than 3 kDa (which was isolated by ultracentrifugation) showed lower IgE and IgG binding capacity compared all other fractions, which contained peptides of higher molecular weight [39]. Thus, allergenic peptides have a higher molecular weight than 3 kDa. This is in line with literature, as previously a molecular weight of at least 3.5 kDa was considered to be necessary to induce an antibody response [9].

Egg proteins seem to be readily digested by different types of proteases [92]. In a mouse model, animals were protected from OVA- and ovomucoid-specific IgE production if they were pre-fed with non-digested or two of the digested egg whites (pepsin digestion or Thermoase PC10F digested). OVA or ovomucoid sensitized mice did not experience a drop of body temperature when challenged with the pepsin or Thermoase OC10F digested egg white, while animals sensitized with non-digested egg white showed a significant drop of body temperature. These results are in line with our study indicating a reduced OVA sensitization capacity and allergic symptoms by support of gastric digestion via gastric enzyme supplementation [81].

### Peanut Allergens

Peanut allergy has a high prevalence throughout all age groups [85] and is known to be associated with severe reactions. Peanut is rich in allergens (> 75% of total proteins) [93], and currently, 17 peanut allergens are described on the official Allergen Nomenclature website by the WHO/IUIS Allergen Nomenclature Sub-Committee (June 2023, http://www.allergen.org/). The major peanut allergens Ara h 1 and 3 belong to the cupin family, while Ara h 2, 6, and 7 are 2S albumins. Ara h 9, 16, and 17 are nsLTPs; Ara h 5 is a profilin; and Ara h 8 is a PR-10 protein [94].

Ara h 2 and Ara h 6 are reported to be digestion stable [95]. Smits et al. examined the effect of digestion and transport across the epithelial layer on the protein allergenicity. The peanut allergens Ara h 1, 2, 3, and 6 were first digested by pepsin and then evaluated for transport across a pig intestinal epithelium. After the transport, the immunoreactivity was assessed by a basophil activation test and a human mast cell activation assay. In contrast to the transported Ara h 2 and 6, the transported Ara h 1 and 3 did not activate basophils. Digested and subsequently transported Ara h 1 and 3 were able to activate mast cells, with the degree of activation depending on the digestion time. Ara h 2 and 6 activated mast cells independent of digestion prior to transport. The authors concluded that digestion and transport affected the allergenicity of Ara h 1 and 3, but not of Ara h 2 and 6 [96].

Processing is able to change the stability of peanut allergens, as was demonstrated by several studies. While thermal processing (without pressure) does not seem to significantly alter the allergen structure or content in peanuts [47], the highly stable Ara h 6 was observed to be irreversibly denaturated after autoclaving significantly decreasing the IgE-binding capacity [97]. Another study reported similar results for Ara h 1 and 3, where a combined treatment with high temperature and pressure (i.e., autoclaving) und subsequent gastro-duodenal digestion reduced the protein content and immunoreactivity [45]. Roasting of peanuts has been attributed an enhanced allergenicity and an increased resistance to digestion [98]. Recently, the opposite was shown and an enhanced digestibility of roasted peanut proteins was reported [99]. The difference may lie in the setup of the simulated digestion, as the study by Di Stasio used an oro-gastroduodenal digestion model including brush border enzymes, while other studies did not include oral or brush border digestion. The authors reported the digestion by brush border enzymes reduced allergenicity of roasted peanuts compared to the raw peanuts, which suggests that brush border peptidases contribute to destroy epitopes of peanut allergens [99].

Not only physical processing methods but also extraction method seems to influence the allergenicity of peanut proteins. Additionally, thermal processing is able to influence the extraction itself, as more proteins could be extracted from raw peanut compared to boiled, roasted, or fried peanut. Different extraction methods influence digestibility and allergenicity of peanut proteins [47]. Moreover, the food matrix also has to be considered when testing allergen stability.

### Shellfish Allergens

The prevalence of shellfish allergies is higher in adults than in early life, and many patients report an adult-onset of shellfish allergy [85]. A variety of species from two different phyla are combined under the term “shellfish,” but the main culprits for allergy belong to the subphylum of Crustacea (such as shrimp, crab and lobster) or the phylum of Mollusca (e.g., oyster). The main, highly cross-reactive allergens found in shellfish are the tropomyosin (recently reviewed in [100]) and the arginine kinase. Other shellfish allergens include myosin light chain, sarcoplasmic calcium-binding protein and troponin C [101].

Tropomyosins are reported to be heat stable [100]. A recent study observed that roasting alone is not sufficient to reduce allergenicity of the shrimp proteins, while heat in combination with reverse-pressure-sterilization was able to reduce allergic symptoms in vivo [102].

Another processing method reported to reduce shrimp allergenicity is microwaving. Tropomyosin levels were reduced up to 75% in microwaved samples compared to the control samples, with higher processing temperature and longer microwaving duration leading to an enhanced reduction [103].

The digestibility of tropomyosin is highly dependent on the digestive conditions. While tropomyosin was susceptible to digestion at low pH (< 2.5), it was highly resistant to pepsin digestion at pH 4 [78].

### Fish Allergens

Most fish allergic patients recognize parvalbumin, especially β-parvalbumin, a calcium-binding protein found in fish muscle. Parvalbumin is found in several fish species in varying concentrations, resulting in different levels of allergenicity of fish [104]. Other fish allergens are e.g., enolases, aldolases, collagen, and tropomyosin [105].

Parvalbumin is generally reported to be resistant to processing and digestion, due to amyloid structures in a B cell epitope of parvalbumin that confer protease resistance [106], but the evidence on the digestibility of parvalbumin is inconclusive. We reported that simulated digestion with lower pH (under 2.5) can rapidly degrade codfish proteins, while this was not observed for pH 2.75 and higher. Also, higher digestion times resulted in a reduction of IgE-binding capability and allergenicity [107]. A recent study supported our findings showing that carp parvalbumin (Cyp c 1) was rapidly digested at pH 1.2 and 2.5 but not at pH 4.0 [78]. A recent study investigated the stability of parvalbumin in European seabass and gilthead seabream. In both species, parvalbumin was detectable after 120 min of gastric digestion. After additional 120 min of intestinal digestion, no parvalbumin could be detected in European seabass digests but low amounts could be still detected in gilthead seabream samples [34].

Recent studies demonstrated that certain processing methods could reduce parvalbumin levels in certain fish species. On a structural level, it was shown that parvalbumin was not able to completely return to its original structure after heating, due to the loss of α-helices [34]. While boiling, ultrasonication and ultraviolet irradiation treatment of parvalbumin from the Japanese scad could not reduce immunogenicity in a mouse model, a combination of Maillard reaction with pressure treatment reduced the immunogenicity of parvalbumin [108]. Steaming was reported to reduce parvalbumin levels in European seabass and gilthead seabream compared to raw samples. Also, freezing at − 20 °C was described to reduce parvalbumin levels in these fish species [34]. Fish muscle can also be further processed into seafood substitutes. The processing into surimi was reported to reduce amyloid aggregation and protease resistance, the overall β-parvalbumin content, and specific-IgE binding. Thus, seafood substitutes made from fish are potentially tolerable to fish allergic patients [109].

The food matrix also seems to have a high impact on the allergenicity of digested parvalbumin, as was shown in a study comparing parvalbumin in lipid emulsion versus non-emulsified parvalbumin. When RBL cells were incubated with the digests of emulsified parvalbumin, higher release of inflammatory mediators was seen and mice were more allergic upon treatment with digests of emulsified parvalbumin [110].

Therefore, the breakdown of parvalbumin might depend on the fish species, the specific processing method, digestive conditions, and the food matrix.

### Carbohydrate Structures as Allergens

Carbohydrate epitopes are described as target for IgE antibodies and able to induce allergic reactions. Following groups of carbohydrates epitopes are described as allergens: (A) classical cross-reactive carbohydrates determinants (N-glycans), (B) mammalian non-human oligosaccharides, (C) O-glycans, (D) glycans from nematode parasites, and (E) galacto-oligosaccharides [111]. Recently, S. P. Commins [112], M. Hils et al. [113], and T. A. Platts-Mills et al. [111] reviewed the role of carbohydrates as allergens.

A prominent glycan epitope is galactose-α-1,3-galactose (α-gal), which is the trigger for red meat allergy. α-gal is foreign to the human glycan repertoire, but it is found in red meat and ticks, which seem to be the sensitizing route. Red meat allergic patients experience delayed clinical symptoms after consumption of red meat (as reviewed here [114]). A study on the digestibility of the α-gal carrying protein bovine thyroglobulin revealed that α-gal retained its allergenic activity after digestion [115]. This was confirmed in another study, where α-gal glycosylation was also reported to lead to altered protein digestion of the carrier protein compared to not-glycosylated proteins. Furthermore, α-gal was reported to hamper the transcytosis of the carrier protein through a Caco-2 monolayer. The authors suggested this as a reason for the delayed onset of red meat allergy [116].

Other carbohydrates with relevance in food allergy are the N-glycans in pineapple bromelain (Ana c 2) and celery (Api g 5) [117] and tomato (Sola l 2). However, the role of cross-reactive carbohydrate determinants (CCD) in clinical reactions towards food is matter of debate since many years [111]. N-glycolyl neuraminic acid, which can be found in milk and meat (Ret D et al., manuscript in preparation), and galacto-oligosaccharides (GOS) in prebiotics might also be relevant, but elucidating their role as allergens needs further research efforts [111]. Ferreira-Lazarte et. al. suggested to include carbohydrases in digestion models, as they are mostly neglected in digestion experiments [118].

## Discussion

In this review, we presented recent evidence on the impact of processing and digestion on allergen stability, showing the importance of combining one or several processing methods with simulated oro-gastrointestinal digestion for simulating a variety of conditions. Overall, harsher processing conditions (e.g., higher temperature, longer processing times) seem to alter allergenicity more efficiently. While higher fragmentation can lead to reduced allergenicity, the fragmented particles may retain the potential to be immunologically active. However, the evidence is often controversial due to differences in experimental setup.

This leads to the question, whether it is possible to establish a generally relevant model for testing allergen stability. In our opinion, personalized models might be clinically more relevant for assessing the protein allergenicity with focus on food allergic patients. Models simulating digestive conditions in food allergic patients of different ages or patients with special medical conditions might help elucidating the mechanisms behind sensitization and clinical manifestations of food allergy.

Moreover, processing and digestion should be evaluated together, potentially even in the context of specific food matrix. We recommend considering food matrix, pre-digestive processing, extraction method, and digestive conditions (oral, gastric, intestinal) when planning allergen stability experiments for risk assessment.

## Conclusion and Outlook

Summarizing the currently available evidence, knowledge regarding stability of allergens against processing and digestion is of relevance in food allergy:

1. Food processing (thermal processing, enzymatic processing, pressure processing, glycation, autoclaving, fermentation etc.) affects the allergenicity-influencing susceptibility to in vitro simulated digestion of a food protein.
2. The digestibility and grade of digestion of allergens is dependent on the experimental conditions (such as pH, time, enzymes used). Therefore, digestive conditions in individual organisms need to be considered when assessing the degree of digestion along the gastrointestinal tract.
3. Even though digestion affects protein allergenicity, it is currently not possible to predict sensitization capacity and allergenicity of allergens reliably based on inherent protein stability to individual influences.

What are the clinical implications of knowledge on allergen stability? In this context, molecular testing is of importance for care of food allergic patients [119]. The identification of relevant allergen molecules may allow precise and personalized recommendations, including information on food processing to decrease allergenicity, if the knowledge is available.

Food allergy is a highly individual disease, as every patient’s specific IgE repertoire recognizes a combination of different allergens. Moreover, the ingestion of allergens can trigger a variety of symptoms. Currently, we have no commonly available treatment option for food allergic patients. It is not possible for clinicians to predict, whether certain processed foods might be tolerated by the patient and possibly even help to re-establish tolerance. Therefore, clinical studies, in which predictive biomarkers and algorithms are researched, are urgently needed to implement the knowledge gained from allergen stability experiments in clinical care of food allergic patients.
